# Wapello County Medical Society

**Published:** 1855-09

**Authors:** H. Kirkpatrick, J. Williamson

**Affiliations:** Pres’t.; Sec’y.


We most cheerfully give place to the following proceedings, not only because they have been the first doings of a county organization we have been solicited to notice, but because of the lively interest which their perusal has awakened and the highly commendable steps taken by our medical brethren in Wapello County.—The course pursued by them reflects much credit and honor upon those who have thus organized themselves into a society, exhibiting a praise-worthy determination to elevate the standard of our noble profession, and a commendable spirit of progress and scientific improvement. It plainly shows too that they are men worthy of their high calling, and that they honor it by their efforts to improve and advance.

These proceedings show that as there is no want of zeal and spirit among the medical men of the West; there are acquirements also. To meet with these gentlemen and note their discussions there would be found enough to sustain the position which we assumed in our last, that in proportion there was as much acquirement as is found in the older States throughout the country, with infinitely more zeal in the cause of medical science and a greater desire to advance in improvement.

### WAPELLO COUNTY MEDICAL SOCIETY.

Pursuant to a call, the following named physicians of Wapello Co., met at Ottumwa, May 26th, for the purpose of organizing a County or District Medical Society.

| | |
|---|---|
| H. Kirkpatrick, | W. Gutch, |
| F. G. McClintick, | J. L. Taylor, |
| A. C. Olney, | T. J. Douglass, |
| J. J. Ellison, | J. L. Laforce, |
| J. C. Kinsey, | A. Hawkins, |
| A. D. Wood, | C. C. Warden, |
| A. K. Weir, | J. Williamson, |

Dr. H. Kirkpatrick was called to the Chair, and Dr. J. Williamson appointed Secretary.

On motion of Dr. A. D. Wood, a committee of three was appointed to draft a Preamble and Resolutions expressive of the object of the meeting. Drs. A. D. Wood, J. C. Kinsey, and W. Gutch were appointed said committee, whereupon the following Preamble and Resolutions were reported and adopted.

#### PREAMBLE AND RESOLUTIONS.

Whereas, Being desirous of sustaining the character of the Medical profession in the Des Moines valley, and co-operating with the Iowa State Medical Society in its efforts to regulate, uphold, and elevate the standard of the profession throughout the State; therefore,

Resolved, 1st, That we organize an association to be called the Wapello County Medical Society.

Resolved, 2d, That we invite and urge upon the physicians of adjoining counties the necessity of co-operating with us in our efforts to promote the diffusion of medical knowledge among the profession, and elevate our calling to that standard of usefulness and respectability which its importance demands.

Resolved, 3d, That the public demand of us an organization of this kind, whereby they may be more fully protected from the impositions of the ignorant and designing. That our profession being one of philanthropy, benevolence, and mercy, and not inferior to any other calling, it should be exalted high, so that empiricism, the low subterfuge, resorted to by quacks only, may find no resting place in this portion of our country; and in forming ourselves into a society, we will have a concert of action and harmony of feeling among its members, the effects of which will be a laudable influence that will and ought to be impressive.

A. D. WO D, Chairman,
WILLIAM GUTCH,
J. C. KINSEY.

On motion, Drs. Kinsey, Wier, Taylor, Gutch, and Douglass, were appointed a committee to draft and report a Constitution and By-Laws for the government of the Society.

Drs. Wood, Ellison, and Hawkins were appointed a committee to report a Fee Bill at the next regular meeting of the Society.

A committee was appointed to nominate permanent officers for the ensuing year. Committee recommended the following, who were elected.

C. C. WARDEN, President.
H. KIRKPATRICK, Vice President.
J. WILLIAMSON, Secretary.
T. J. DOUGLASS, Cor. Sec'y.

A. D. WOOD,

W. GUTCH,

J. C. KINSEY,

A. K. WEIR,

J. L. TAYLOR,

} Board of Censors.

The Constitution declares that the Society shall be called the Wapello County Medical Society—that its object shall be to promote the diffusion of knowledge, elevate the standard of the profession and promote unanimity of feeling and concert of action among its members. It provides for the election of members of good moral character who have passed a satisfactory examination before the board of Censors,—that the officers shall be a President, Vice President, Secretary, Cor. Secretary, and Treasurer,—that the annual meeting of the Society be on the first Saturday of May in next year.

The By-Laws provide that the regular meetings of the Society he held on the fourth Saturdays of May, Aug., Nov., and Feb.—They provide for the election of officers annually by ballot,—that eight members shall constitute a quorum for the transaction of business at any regular meeting.

Provision is also made for the reading of at least one dissertation on some Medical or Scientific subject at each regular meeting, also it is made the duty of each member of the Society to preserve a faithful record of each important case that comes under his charge, together with the treatment thereof, and report the same to the Society at its next regular meeting thereafter.

President appointed Dr. J. Williamson to prepare and deliver an essay on some medical or scientific subject at the regular meeting in August next.

Ordered that the Secretary furnish a report of the proceedings to the Iowa Medical Journal for publication.

June 9th.—First regular meeting, Drs. S. G. Norris and S. P. Johnson, upon application, were recommended and voted members of the Society. Drs. Kinsey and Kirkpatrick were appointed delegates to the meeting of the Iowa S. M. S., 14th inst.

H. KIRKPATRICK, Pres't.

J. WILLIAMSON, Sec'y.

